# Application of microsublimation for sample purification in compound-specific radiocarbon analysis

**DOI:** 10.1186/s40645-025-00770-y

**Published:** 2025-10-30

**Authors:** Christian Heusser, Caroline Welte, Lukas Wacker, Kai Sebastian Nakajima, Thomas M. Blattmann, Negar Haghipour, Timothy Ian Eglinton

**Affiliations:** 1 Geological Institute, ETH Zürich, Sonneggstrasse 5, 8092 Zurich, Switzerland; 2 Laboratory of Ion Beam Physics, ETH Zürich, Otto-Stern-Weg 5, 8093 Zurich, Switzerland; 3 PSI Paul Scherrer Institut, Forschungsstrasse 111, 5232 Villigen, Switzerland; 4 ETH Bibliothek, ETH Zürich, Rämistrasse 101, 8092 Zurich, Switzerland; 5 Asian School of the Environment, Nanyang Technological University, 50 Nanyang Avenue, Singapore, 639798 Singapore

**Keywords:** Radiocarbon analysis, Sublimation, Sample purification, AMS, ^14^C

## Abstract

This article presents the development and application of a microsublimation apparatus aimed at improving the purity of ultra-small samples for compound-specific radiocarbon analysis. Accurate radiocarbon (^14^C) measurements require the effective isolation of biomarkers, yet procedural steps, such as chromatography and sample transfer, introduce contamination risks that can skew results. Here, we present a novel approach to remove contamination resulting from chromatographic isolation. The apparatus, constructed primarily from aluminum, allows solvent-free sublimation of multiple samples under vacuum. A constant contamination assessment showed a blank mass of 1.35 µg of carbon with a F^14^C of 0.33, indicating minimal contamination with ^14^C-depleted carbon. The apparatus demonstrated high efficacy for compounds with higher melting points, such as amino acids and dyes, while compounds like alkanes showed lower recovery rates. These findings confirm the potential of microsublimation to enhance post-chromatography sample purity and improve the accuracy of ^14^C measurements, though challenges remain for certain compound classes.

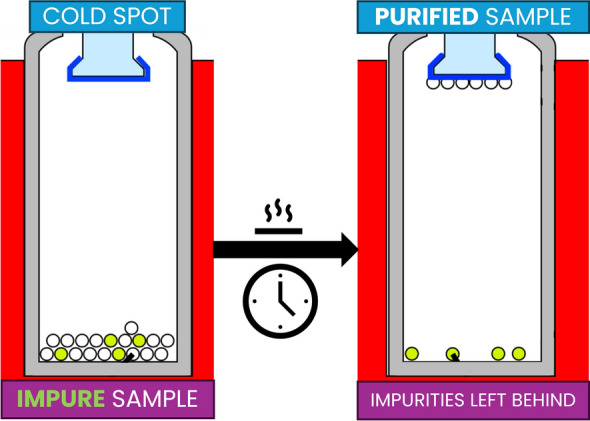

## Introduction

Obtaining precise ^14^C measurements in CSRA necessitates the meticulous extraction and isolation of individual biomarkers. These processes, often conducted via preparative capillary gas chromatography (PCGC) or preparative high-performance liquid chromatography (prep-HPLC), involve numerous intermediate steps, such as filtration, desalting, drying, and sample transfer. Each of these steps carries a risk of introducing contaminants that can add extraneous carbon to the sample, thereby affecting the final ^14^C measurement. Sources of contamination include labware, solvents, and column bleed from chromatographic equipment. While the contamination from any single step may be negligible, the cumulative effect across the entire procedure can lead to significant deviations in the measured ^14^C content, potentially distorting the interpretation of biomarker data (e.g., Casanova et al. 2020; Shah and Pearson 2007; Reetz et al. 2024; Hanke et al. 2017).

Procedural blanks are routinely assessed to account for extraneous carbon and correct the ^14^C measurements. However, even with blank correction, further methodological advancements are essential to minimize contamination, particularly for small sample sizes. Improvements in sample handling are often biomarker-specific and require significant effort to optimize (Haghipour et al. 2019). Post-isolation contamination remains a critical issue, particularly from solvents used for sample transfer and metal vessels for sample containment, both of which introduce carbon that can alter the radiocarbon signature of the sample (Welte et al. 2018).

Several approaches have been proposed to address these contamination sources. For example, Casanova et al. (2018) developed a modified PCGC technique in which samples are trapped directly on glass wool within capillaries, eliminating the need for solvents during transfer. Although this method significantly reduces contamination during PCGC, it does not address column bleed and is limited to biomarkers isolated by this specific chromatographic technique. Furthermore, comparable improvements for prep-HPLC are very limited and tailored for specific biomarkers (e.g., Ishikawa et al. 2018; Blattmann et al. 2020).

Sublimation is a well-known purification technique that may offer a viable solution for the solvent-free purification and transfer of isolated samples for CSRA. Sublimation involves the direct phase transition of a solid to a gas, bypassing the liquid phase. This technique has been used extensively for the purification of organic and inorganic compounds (e.g., Karpinska et al. 2011; Refat et al. 2024; Seo et al. 2002; Liu et al. 2008). Additionally, sublimation has been applied under extreme conditions to purify sensitive biomolecules such as amino acids and nucleic acids, as demonstrated in studies by Glavin and Bada (1998), Glavin et al. (2002). These studies showed that amino acids and nucleic acids can undergo sublimation at very high temperatures, enabling their purification while preserving their chemical integrity.

**Fig. 1:**
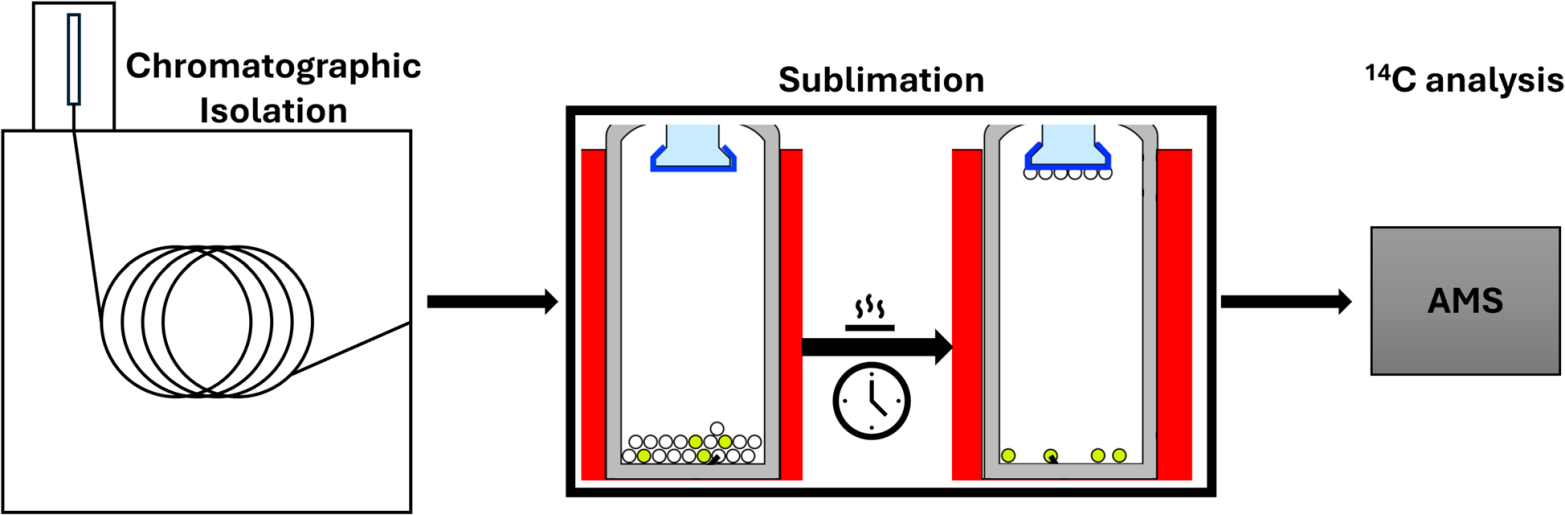
Schematic representation of the sample preparation workflow

In this article, we introduce and explore the application of microsublimation as a purification step following chromatographic isolation in CSRA sample preparation. A schematic illustration of the workflow is shown in Fig. 1. This method not only serves as an effective approach for purifying samples and minimizing contamination but also offers a promising technique of transferring samples into metal vessels for ^14^C analysis. For post-chromatographic purification of isolated compounds, sublimation allows for the removal of contaminants without the need of solvents, which are often a source of background noise in CSRA. By directly transitioning compounds from a solid to a gaseous state, sublimation can enhance the purity of isolated compounds and contribute to more accurate ^14^C measurements.

## Methods/experimental procedures

### Microsublimation apparatus design

**Fig. 2:**
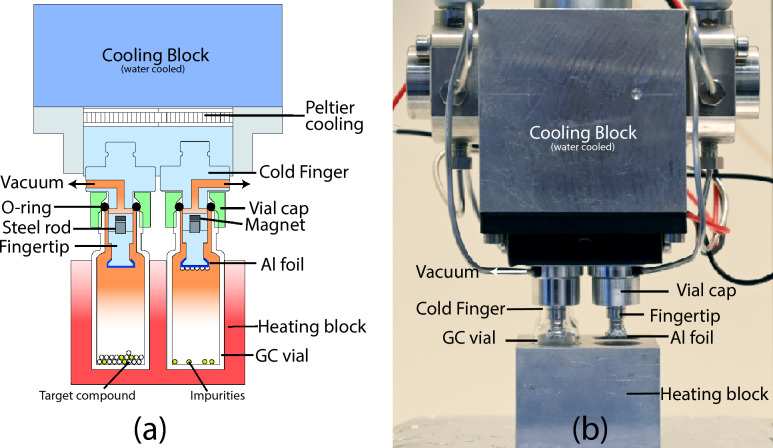
Microsublimation apparatus design. **a** Sketch of the microsublimation apparatus, with starting (left vial) and final (right vial) conditions. **b** Photograph of the microsublimation apparatus (*Credits:* Stephan Wartenweiler)

The microsublimation apparatus used is derived from an existing glass prototype (Heusser 2020), is primarily constructed from aluminum (Al, type: Anticorodal 112), and consists of three main components: the cold fingers, including the cooling block, the sample vessel, and the vessel holder/heating block. A sketch and photograph of the sublimation apparatus are shown in Fig. 2. The dimensions of the key components are as follows: the cold fingers have a diameter of 5 mm, the heating block measures 3.5 cm by 3.5 cm by 2.5 cm, and the cooling block has dimensions of 5 cm by 5 cm by 5 cm.

The cold finger assembly is subdivided into cold finger bases with detachable tips, which are held together magnetically. This design is achieved by embedding a magnet into the cold finger base and a steel rod into the fingertip. Additionally, the vertical offset of both the magnet and the steel rod prevents horizontal displacement, thereby stabilizing the assembly. Cooling of the cold fingers is accomplished using Peltier elements, which generate a temperature differential between their hot and cold sides. The hot side is connected to an Al block (cooling block) that is actively water cooled to dissipate the heat. The cooling block also includes valves for the vacuum line, allowing the system to be evacuated. The apparatus is designed to support four cold fingers, enabling the simultaneous sublimation of multiple samples onto the cold fingertips.

The sample vessels used in this apparatus are ND9 threaded glass vials, commonly known as GC vials. This choice reduces the need for sample transfer and offers a flexible solution for various experimental requirements. The vials are secured to the cold fingers with custom aluminum vial caps, and the system is sealed gas-tight using Viton O-rings.

The vessels are heated on a hot plate in an Al heating block that ensures uniformal heating of the glass surfaces. The design minimizes the undesired deposition of the sublimate on cold walls and ensures a high recovery on the cold finger.

### Materials

For the fabrication of Al capsules, we used Tangan No 42 Al foil (thickness ca. 13 µm) which was purchased from Migros. For sublimation experiments, *n*-C_28_, *n*-C_32_, l-glutamic acid (Glu), l-valine (Val) (^14^C modern and dead), l-phenylalanine (Phe), l-, dl-tryptophan, vanillin (^14^C modern and dead), and syringic acid were purchased from Sigma-Aldrich, alizarin was purchased from Acros Organics, and purpurin was purchased from Cayman Chemical. Madder root (*rubia tinctorum*) powder was provided by L. Hendriks (School of Engineering and Architecture of Fribourg, Fribourg, Switzerland).

### General protocol for microsublimation

We established a general protocol that was followed for all measurements. A 10-mm circle of Al foil was wrapped around the bottom of the fingertip and subsequently heat-treated at 450 °C for 6 h to eliminate contamination from extraneous carbon. The fingertip was then attached to the base of the apparatus using a pair of tweezers (pre-cleaned with milliQ water, methanol and dichloromethane). A GC vial containing the sample was then affixed to the apparatus and it was subsequently evacuated while monitoring the pressure (target value: 0 mbar) to ensure the apparatus remained sealed.

The apparatus was submerged into the heating block and the cooling was turned on. After about one minute, the hotplate was turned on. In cases where there were volatile contaminants, the hotplate was turned on prior at a lower-temperature set to remove said contaminants (Note: the use of Peltier elements also allows to heat the cold fingers by reversing the direction of the current, which can promote contamination removal).

The sublimation progress was regularly checked by visual inspections of the cold fingers and GC vials. Upon completion of sublimation, the hotplate was turned off and the apparatus was allowed to cool before turning off the Peltier cooling. The cold finger was allowed to heat to room temperature, and any condensation was wiped off before opening the apparatus. The GC vials were then cautiously unscrewed. The removal of the cold fingertips was done using a pair of tweezers. Finally, the fingertip was placed into a 12-mm circle of Al foil, and the cap was loosened and wrapped carefully before being stored in a heat-treated GC vial. Radiocarbon analysis was performed on an elemental analyzer (EA) coupled to a MICADAS AMS system at the Laboratory of Ion Beam Physics, ETH Zürich (Fahrni et al. 2013; Ruff et al. 2010a, 2010b Synal et al. 2007).

The same EA system was used to determine the carbon mass of each sample prior to graphitization. These values were used to calculate the recovery by comparing the EA-measured carbon mass to the initial amount of compound carbon introduced into the microsublimation unit.

### Constant contamination assessment

Data processing was done using the in-house BATS software. In all cases, radiocarbon measurements are reported as F^14^C, which is the ratio of the radiocarbon content in a sample to that of a modern reference standard based on atmospheric carbon dioxide levels in 1950. We applied the model of constant contamination for the evaluation of the mass m_c_ and F^14^C of extraneous carbon (F^14^C_c)_ and extraction of the F^14^C of samples (F^14^C_s_) from the AMS measurements (F^14^C_m_) (Hanke et al. 2017).

$$F^{14} C_{s} = \frac{{F^{14} C_{m} {*}m_{m} - F^{14} C_{c} {*}m_{c} }}{{m_{m} - m_{c} }},$$

where *m*_*m*_ is the total measured *C* mass, which is the sum of the actual *C* mass of the sample *m*_*s*_ and of the contamination *m*_*c*_.

The corresponding uncertainty of F^14^C_s_ is derived from error propagation:

$$\begin{aligned} \sigma_{{F^{14} C_{s} }}^{2} & = \left[ {\sigma_{{m_{c} }} \left( {\frac{{F^{14} C_{m} {*}m_{m} - F^{14} C_{c} {*}m_{c} }}{{\left( {m_{m} - m_{c} } \right)^{2} }}} \right) - \left( {\frac{{F^{14} C_{c} }}{{m_{m} - m_{c} }}} \right)} \right]^{2} \\ & \quad + \left[ {\sigma_{{m_{m} }} \left( {\frac{{F^{14} C_{m} }}{{m_{m} - m_{c} }} - \frac{{F^{14} C_{m} {*}m_{m} - F^{14} C_{c} {*}m_{c} }}{{\left( {m_{m} - m_{c} } \right)^{2} }}} \right)} \right]^{2} + \left[ {\sigma_{{F^{14} C_{m} }} \frac{{m_{m} }}{{m_{m} - m_{c} }}{ }} \right]^{2} \\ & \quad + \left[ {\sigma_{{F^{14} C_{c} }} \frac{{ - m_{c} }}{{m_{m} - m_{c} }}} \right]^{2} . \\ \end{aligned}$$

The correction for constant contamination was done using ^14^C dead Val (F^14^C = 0.01) and ^14^C modern Val (F^14^C = 1.06).

## Results

### Design iterations

The existing prototype (Heusser 2020) was constructed primarily from glass and consisted of two main components: the cold finger part and the vessel containment part. These components were connected using a ground glass joint, with a Teflon ring employed to achieve a grease-free seal. The cold finger part was uniquely designed with an indented feature that enabled the fixation of metal capsules. The glass material allowed the prototype to undergo heat treatment, which enhanced the quality of the capsule fixation process. The cold finger was water cooled and included a valve for evacuation. The vessel containment part was designed to accommodate a GC vial containing the sample, though it also allowed for direct sample insertion into the containment unit.

The initial testing of the prototype yielded promising results and demonstrated the potential of microsublimation. However, testing also revealed several issues and limitations that informed subsequent design changes. The prototype allowed for only one measurement per cycle, followed by a necessary heat treatment of the entire apparatus (6 h at 450 °C) and introducing significant inefficiencies. Since glass was the primary material used in this early design, it presented limitations in downsizing, as reducing its dimensions introduce stress and significantly increase the risk of material failure. Consequently, large metal capsules with a diameter of 25 mm were needed, limiting the potential for miniaturization.

Cooling requirements also presented challenges, as water cooling required a chiller and precise flow adjustments to prevent the capillary within the cold finger from bursting. Additionally, the coolant temperature was relatively high, maintained at approximately 18 °C (due to chiller limitations), which further limited cooling efficiency. Using coolant additives to reach lower temperatures, such as glycerol or ethylene glycol, was not feasible due to increased viscosity and high carbon content, which posed a risk of charring during heat treatment.

To address the limitations of the glass apparatus prototype, we designed an improved version that retained the desired features while overcoming the identified drawbacks. The new apparatus was constructed from aluminum, chosen for its excellent machinability, heat tolerance, and thermal conductivity. This material selection also enabled the design to incorporate four cold fingers arranged in a 2 × 2 configuration, allowing for simultaneous sublimation runs. A significant change in the new design was the transition from a sample containment unit to the direct attachment of a GC vial to the cold finger, simplifying the overall setup.

We replaced the water-cooling system of the cold finger with Peltier element cooling, which not only reduced space requirements but also improved cooling power, achieving to maintain low temperatures of the cold finger during sublimation experiments. The Peltier elements were mounted on an aluminum block, which also served as the attachment point for all necessary valves and connections. To facilitate evacuation, a small hole was drilled into each cold finger, which was then connected to a vacuum pump via valves and capillaries. Initial tests revealed that the aluminum block’s heat capacity was insufficient to maintain stable cold finger temperatures over prolonged time periods. This issue was resolved by drilling a channel into the block, enabling water cooling to dissipate the heat generated by the Peltier elements. Despite going back to water cooling, this solution still represented an improvement over the glass prototype, as the water cooling of the aluminum block only required sufficient water flow to dissipate heat, without the need for precise flow adjustments. Additionally, the Peltier cooling system achieved lower temperatures than the previous water-cooling method, further enhancing the apparatus’s performance.

The cold finger part underwent several design iterations to address the challenges encountered during testing. The first version featured a single-part cold finger with a small indentation to hold the Al foil, which was not heat-treatable. This required the meticulous attachment of a heat-treated aluminum tip to the cold finger. The small indentation led to weak capsule fixation, necessitating the use of a thin copper wire to secure the capsule.

In response to these issues, we redesigned the cold finger, splitting it into two parts: a cold finger base housing the vacuum connection and a fingertip with a larger indentation. The fingertip was attached to the base using a threaded connection, allowing it to undergo heat treatment, thereby improving capsule fixation. However, the process of connecting the fingertip to the base proved to be tedious and time-consuming.

To simplify this process, we introduced two additional indentations that allowed the fingertip to be securely held in a wrench, significantly easing the attachment to the base. Despite this improvement, careful handling was still required, as the threads were prone to irreparable damage if not aligned correctly, leading to the loss of two cold finger bases within 10 runs. After considering various solutions, including the replacement of the Al cold finger base with harder and more durable beryllium bronze (CuBe), we ultimately decided to explore alternative attachment methods.

We found that a magnet-based attachment system offered a simple yet effective solution. However, maintaining the heat treatability of the fingertip while using a magnet posed a challenge, as magnets lose their permanent magnetic properties above the so-called Curie temperature, which in the case of Nd magnets is at temperatures above 300 °C (Rumble et al. 2025). We resolved this by inserting a steel rod into the fingertip that is heated for cleaning and using a recessed NdFeB magnet in the base, which is never heated. The steel rod was allowed to protrude slightly to prevent horizontal displacement of the fingertip. This final iteration resulted in a sublimation apparatus that was user-friendly and capable of producing reproducible results (Fig. 2).

### Operational parameters

#### Vacuum requirement

Our results demonstrate that maintaining a vacuum is crucial for the efficient progression of the sublimation process. In the absence of a vacuum, sublimation was either significantly hindered or entirely prevented. This was particularly evident in samples that were inadequately sealed, leading to pressure readings exceeding 5 mbar, or in those exposed to a controlled helium (He) atmosphere. In both scenarios, even after extended exposure times, little to no sublimation occurred. These observations suggest that the presence of gas impedes not only the phase transition from solid to gas but also the transport of the compound to the cold finger. Furthermore, in non-vacuum conditions, reduced cooling power was observed, indicated by the melting of condensed ice on the outer walls of the vial caps and cold finger bases.

Additionally, for samples contained in unsealed vials, visible signs of oxidation were observed, as indicated by a pronounced darkening of the material. This oxidation not only suggests that atmospheric oxygen interfered with the sublimation process but also underscores the necessity of vacuum conditions to prevent sample degradation during purification.

These observations emphasize the critical role of a vacuum in both facilitating sublimation and preserving the integrity of the sample, ensuring that the material remains unaltered throughout the process.

#### Temperature dependence

We conducted a set of experiments to investigate temperature requirements and conditions for sublimation, focusing on lower-temperature sublimation techniques compared to previously used high-temperature methods. For amino acids, we examined the sublimation behavior of Val, which has one of the highest melting points (298 °C) among proteinogenic amino acids.

**Fig. 3:**
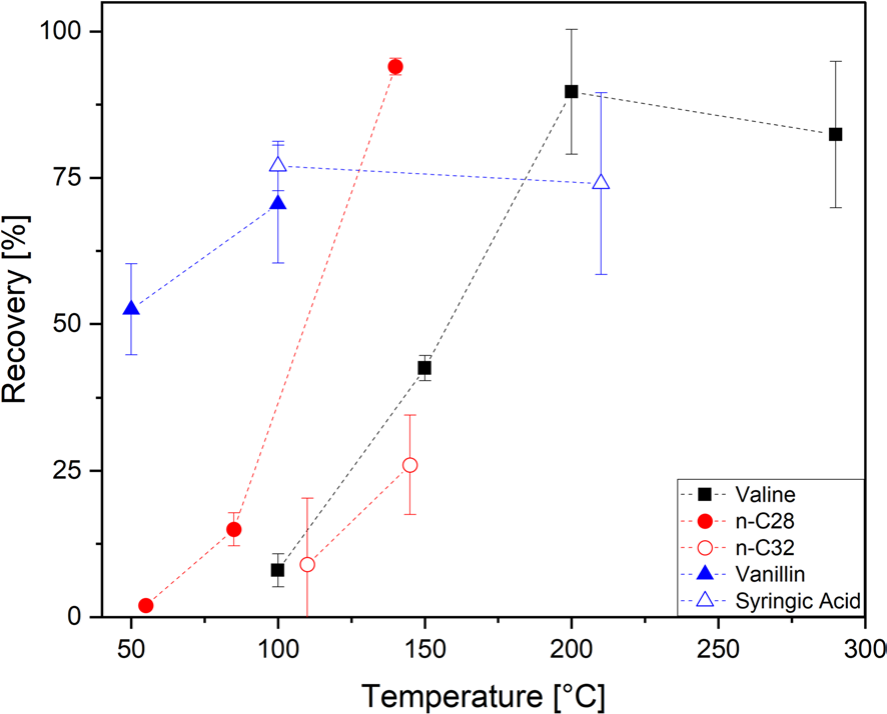
Sublimation recoveries for different compounds at various temperatures. The compounds shown are amino acid Val (black filled square), *n*-alkanes *n*-C_28_ (red filled circle) and *n*-C_32_ (red opened circle), and lignin phenols vanillin (blue filled triangle) and syringic acid (blue opened triangle)

Val samples were incrementally heated on a hotplate to test for sublimation at different temperatures. We tested recoveries at temperature settings of 100 °C, 150 °C, 200 °C, and 290 °C (Fig. 3). At 100 °C and only after a prolonged sublimation duration of 120 min, a limited degree of sublimation was observed, with recoveries recorded at 8 ± 3%. Sublimation efficiency improved notably at 150 °C, where recoveries increased to (43 ± 2%) after the same duration. At 200 °C, sublimation was near-complete, yielding a recovery of (90 ± 11%), although this temperature remained significantly below the melting point of 298 °C. A further increase to 290 °C resulted in slightly reduced recoveries (82 ± 13%), likely due to alternate sublimation dynamics at this higher temperature.

For *n*-alkanes, we tested *n*-C_28_ and *n*-C_32_ homologues, which have similar melting points (*n*-C_28_: 57 °C, *n*-C_32_: 65 °C) (Fig. 3). At a temperature of 55 °C, *n*-C_28_ showed no visible sublimation and a recovery of 2%, even after 3 h. Increasing the temperature to 85 °C resulted in a recovery of 15 ± 3% after the same duration. Further raising the hotplate temperature to 140 °C increased recoveries to over 94 ± 1% after 1 h. For *n*-C_32_, a similar but less consistent trend was observed: At 110 °C, recoveries ranged from 9 ± 11% after 2 h. When the temperature was raised to 145 °C, recoveries improved to 26 ± 9%.

For lignin phenols, we tested syringic acid and vanillin. For vanillin (melting point 81 °C), recoveries increased from 52 ± 8% at 50 °C to 70 ± 10% at 100 °C. For syringic acid (melting point 205 °C), recoveries were 77 ± 4% at 100 °C to 74 ± 16% at 210 °C.

### Compatible materials

To evaluate the versatility and applicability of the microsublimation apparatus, we selected a representative set of compound classes commonly targeted in geochemical, archaeological, and environmental studies spanning a range of microsublimation settings. These include amino acids, lignin phenols, long-chain *n*-alkanes, and natural dyes. Each of these compound classes plays a central role in molecular-level investigations of carbon sources, turnover, or provenance, and they are frequently analyzed using CSRA. Amino acids, for example, are increasingly used in archaeological contexts for dating bone collagen or preserved organic residues (e.g., McCullagh et al. 2010) and have potential for applications biogeochemical studies as indicators of microbial input and organic matter composition in soils and sediments (e.g., Blattmann and Ishikawa 2020). Lignin phenols are well-established markers for terrestrial vascular plant material and its degradation state (e.g., Feng et al. 2017). Long-chain *n*-alkanes are used in paleoenvironmental reconstructions due to their plant-wax origin and source-specificity (e.g., Van Der Voort et al. 2017). Natural dyes, such as alizarin and purpurin, are of interest in archaeological studies, where molecular-level purification may help isolate dye components from complex matrices (e.g., Hendriks et al. 2019). These chemically diverse yet application-relevant compounds provide a robust test set for assessing sublimation performance across a range of physicochemical properties.

We tested the sublimation apparatus with a variety of compounds from these compound classes (Table 1), primarily focusing on amino acids such as Glu, Phe, and Val. These amino acids were selected due to their differing melting points (Glu: 207 °C, Phe: 267 °C, Val: 298 °C), covering a broad temperature range typical for amino acids. In general, amino acids have relatively high-melting points, which makes them particularly suitable for evaluating the performance of the microsublimation apparatus. Their physical properties allow testing at elevated temperatures, providing a robust system to assess whether sublimation can proceed cleanly while minimizing the risk of decomposition or oxidation under vacuum conditions. For Val, both ^14^C modern and ^14^C dead standard materials were available, allowing for blank assessments using the same compound.

**Table 1:** Combined list of microsublimation samples

ETH nr	Compound	*T* (°C)	*t* (min)	F^14^C reference	F^14^C measured	*m*_*i*_ (µg)	*m*_EA_ (µg)	Recovery (%)
*Amino acids*								
125305.1.1	Glutamic acid	200	90	1.074	1.066 ± 0.008	47	42	89
125306.1.1	Glutamic acid	200	90	1.074	1.060 ± 0.008	91	77	85
125308.1.1	Phenylalanine	260	105	1.08	1.081 ± 0.008	48	44	92
125309.1.1	Phenylalanine	260	105	1.08	1.066 ± 0.009	35	50	143
127927.1.1	Valine	290	90	1.064	1.042 ± 0.008	51	49	96
127928.1.1	Valine	290	90	1.064	1.026 ± 0.009	23	22	96
127929.1.1	Valine	290	90	1.064	1.002 ± 0.010	24	20	83
127930.1.1	Valine	290	90	1.064	1.036 ± 0.008	49	44	90
125311.1.1	Valine	290	90	1.064	1.036 ± 0.009	34	26	76
125312.1.1	Valine	290	90	1.064	1.174 ± 0.009	59	52	88
125313.1.1	Valine	290	90	1.064	1.059 ± 0.008	65	61	94
127919.1.1	Valine	290	90	1.064	1.002 ± 0.031	5	3	60
127921.1.1	Valine	290	90	0.013	0.023 ± 0.002	40	33	83
127931.1.1	Valine	290	90	0.013	0.017 ± 0.002	44	39	89
127932.1.1	Valine	290	90	0.013	0.038 ± 0.002	25	18	72
127933.1.1	Valine	290	90	0.013	0.018 ± 0.002	50	41	82
127934.1.1	Valine	290	90	0.013	0.024 ± 0.002	26	25	96
126548.1.1	Valine	290	90	0.013	0.054 ± 0.003	14	8	57
126549.1.1	Valine	290	90	0.013	0.017 ± 0.001	42	31	74
134632.1.1	Valine	290	30	1.064	0.965 ± 0.099	29	0.9	3
134639.1.1	Valine	100	120	1.064	1.005 ± 0.096	9	0.9	10
134640.1.1	Valine	100	120	1.064	0.910 ± 0.070	16	1	6
134646.1.1	Valine	200	30	1.064	1.072 ± 0.011	25	23	92
134647.1.1	Valine	200	30	1.064	1.042 ± 0.010	25	29	116
134648.1.1	Valine	200	30	1.064	1.057 ± 0.011	25	23	92
134649.1.1	Valine	200	30	1.064	1.078 ± 0.011	25	24	96
134650.1.1	Valine	200	30	1.064	1.074 ± 0.011	25	21	84
134651.1.1	Valine	200	30	1.064	1.085 ± 0.011	25	21	84
134654.1.1	Valine	150	120	1.064	1.065 ± 0.018	18	8	44
134655.1.1	Valine	150	120	1.064	1.070 ± 0.016	22	9	41
134658.1.1	Valine	200	30	1.064	1.036 ± 0.029	5	3	60
134662.1.1	Valine	200	30	1.064	1.085 ± 0.026	5	5	100
134631.1.1	Valine	290	30	1.064	1.002 ± 0.012	29	10	34
134633.1.1	Valine	290	30	1.064	1.031 ± 0.011	32	16	50
134634.1.1	Valine	290	30	1.064	0.806 ± 0.029	32	1	3
134659.1.1	Valine	200	30	1.064	1.019 ± 0.014	10	0.9	9
134663.1.1	Valine	200	30	1.064	1.017 ± 0.015	10	10	100
134666.1.1	Valine	200	30	1.064	1.027 ± 0.011	20	17	85
134667.1.1	Valine	200	30	1.064	1.033 ± 0.009	40	38	95
134670.1.1	Valine	200	60	1.064	1.033 ± 0.011	20	20	100
134671.1.1	Valine	200	60	1.064	1.034 ± 0.009	40	35	88
134674.1.1	Valine	200	60	1.064	1.023 ± 0.011	20	17	85
134675.1.1	Valine	200	60	1.064	1.036 ± 0.009	40	38	95
134661.1.1	Valine	200	30	0.013	0.097 ± 0.004	10	8	80
134664.1.1	Valine	200	30	0.013	0.071 ± 0.005	5	5	100
134665.1.1	Valine	200	30	0.013	0.052 ± 0.004	10	8	80
134668.1.1	Valine	200	30	0.013	0.704 ± 0.006	20	18	90
134672.1.1	Valine	200	60	0.013	0.021 ± 0.002	20	17	85
134673.1.1	Valine	200	60	0.013	0.014 ± 0.001	40	33	83
134676.1.1	Valine	200	60	0.013	0.026 ± 0.002	20	17	85
134677.1.1	Valine	200	60	0.013	0.016 ± 0.001	40	35	88
121867.1.1	Tryptophan	290	60	0.02	0.021 ± 0.002	–	104	–
121868.1.1	Tryptophan	290	60	0.02	0.012 ± 0.002	–	208	–
121869.1.1	Tryptophan	290	60	0.02	0.008 ± 0.002	–	174	–
121870.1.1	Tryptophan	290	60	0.02	0.009 ± 0.002	––	187	–
*Lignin phenols*								
127917.1.1	Syringic acid	210	45	0.794	0.802 ± 0.010	27	17	63
127918.1.1	Syringic acid	210	45	0.794	0.798 ± 0.007	53	45	85
127915.1.1	Vanillin	100	45	0.995	0.939 ± 0.018	15	9	60
127916.1.1	Vanillin	100	45	0.995	0.983 ± 0.008	47	38	81
127913.1.1	Vanillin	100	60	0.009	0.010 ± 0.002	66	51	77
127914.1.1	Vanillin	100	60	0.009	0.075 ± 0.003	25	16	64
134641.1.1	Syringic acid	100	70	0.794	1.069 ± 0.012	20	16	80
134642.1.1	Syringic acid	100	70	0.794	0.965 ± 0.011	19	14	74
134656.1.1	Syringic acid	150	12	0.794	0.917 ± 0.008	32	44	138
134657.1.1	Syringic acid	150	12	0.794	0.826 ± 0.008	36	29	81
134637.1.1	Vanillin	50	90	0.995	1.007 ± 0.009	151	88	58
134638.1.1	Vanillin	50	90	0.995	1.013 ± 0.009	72	34	47
*Alkanes*								
126551.1.1	*n*-C_28_	55	180	0.003	0.585 ± 0.050	48	1	2
126552.1.1	*n*-C_28_	85	200	0.003	0.480 ± 0.035	15	2	13
126553.1.1	*n*-C_28_	85	200	0.003	0.514 ± 0.018	23	4	17
126554.1.1	*n*-C_28_	140	60	0.003	0.023 ± 0.002	19	18	95
126555.1.1	*n*-C_28_	140	60	0.003	0.009 ± 0.001	41	38	93
127923.1.1	*n*-C_32_	110	120	1.07	0.703 ± 0.056	68	1	1
127924.1.1	*n*-C_32_	110	120	1.07	0.973 ± 0.042	12	2	17
126556.1.1	*n*-C_32_	145	200	1.07	1.030 ± 0.011	74	15	20
126557.1.1	*n*-C_32_	145	200	1.07	1.008 ± 0.012	34	11	32
*Dyes*								
134643.1.1	Alizarin	240	45	Dead	0.011 ± 0.001	118	108	92
134644.1.1	Purpurin	240	45	Dead	0.014 ± 0.001	111	75	68
134645.1.1	R. tinctorum	240	45	Modern	0.954 ± 0.015	112	6	5

*T* temperature, *t* sublimation time, *m*_*i*_ initial sample C mass, *m*_*EA*_ C mass measured by EA

In addition to amino acids, other compound classes were investigated, including lignin phenols, *n*-alkanes and dyes. For lignin phenols, we tested syringic acid and vanillin. We found that both compounds sublimed well; however, recoveries were only around 70%. We observed deposits on uncovered parts of the fingertip after sublimation. Especially for vanillin, this could be further confirmed due to the characteristic odor of the compound.

For *n*-alkanes, as mentioned before, high recoveries were only obtained for *n*-C_28_ at temperatures that were about 90 °C above the melting point. For *n*-C_32_, recoveries remained low even at 90 °C above the melting point. Therefore, we did not find the application of sublimation suitable for these compounds. We hypothesize that the low volatility of these compounds, even at elevated temperatures, contributes to incomplete sublimation. Additionally, weak adsorption to surfaces or recondensation within the vial may lead to apparent losses. Therefore, we did not find sublimation to be a suitable purification method for these compounds.

Dyes, including alizarin (melting point 290 °C), purpurin (melting point 259 °C), and madder root (*Rubia tinctorum*) powder, were also tested. While sublimation of pure alizarin and purpurin was successful, sublimation of madder root was not. The madder root sample charred during sublimation, and only a small fraction sublimed onto the cold finger. One possible explanation for this outcome could be matrix effects, as the complex composition of natural materials like madder root may promote interactions or decomposition reactions that interfere with clean sublimation.

### Removal of impurities by microsublimation

The efficiency of microsublimation in removing contaminants was tested by mixing vanillin with approx. 20 wt% Val or Glu to simulate impurities. Due to the significant difference in melting points between vanillin and the impurities, the experiment aimed to determine whether these contaminants would co-sublime with the target compound. Val, with a melting point about 200 °C higher than vanillin, and Glu, with a melting point approximately 100 °C higher, were selected to assess if the difference in melting points alone could prevent co-sublimation. This choice allowed us to evaluate whether a 100 °C difference, as in the case of Glu, would already be sufficient for separation, and how much more effective the separation would be with a 200 °C difference, as with Val.

Two samples from each mixture were subjected to sublimation. One of the samples was contaminated during the wrapping process after it fell to the floor, which was later confirmed by ^14^C measurements. The ^14^C measurements on the remaining samples showed no evidence of co-sublimation between vanillin and the impurities (Table 2). All samples fell within the error range of the reference values, suggesting that the impurities did not transfer during sublimation.

**Table 2:** ^14^C measurements of mixtures of ^14^C dead vanillin (F^14^C = 0.009) and ^14^C modern amino acids (glutamic acid and valine) as impurity proxies

ETH nr	Impurity	F^14^C
136135.1.1	Ref. mixture	0.106 ± 0.003
136131.1.1	Glutamic acid	0.000 ± 0.002
136133.1.1	Glutamic acid	0.006 ± 0.002
136132.1.1	Valine	0.002 ± 0.002
136134.1.1	Valine^a^	0.108 ± 0.003

Ref. mixture refers to the mixture of amino acids and vanillin prior sublimation

^a^Sample was contaminated after sublimation as it fell to the ground during the wrapping process

### Constant contamination assessment

**Fig. 4:**
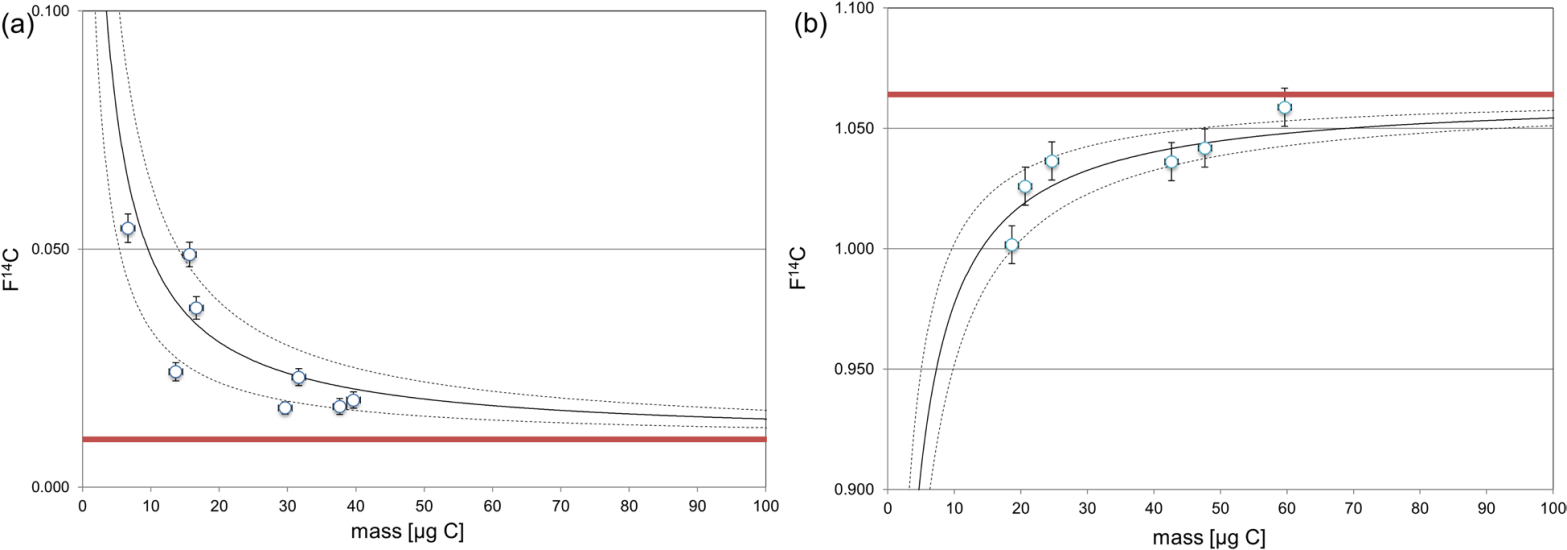
Constant contamination assessment for **a** ^14^C depleted and **b** ^14^C modern Val standard. Fitting of the data points resulted in a blank of 1.35 ± 0.41 μg C with F^14^C = 0.33 ± 0.10. The red lines represent the reference F^14^C value of the ^14^C modern and ^14^C dead standards

The constant contamination assessment was carried out on both ^14^C modern and ^14^C dead Val samples. A reduced dataset was employed, as one batch of AMS measurements was excluded due to elevated F^14^C values in the modern samples, indicating potential anomalies. The final dataset included 8 ^14^C dead samples and 6 ^14^C modern samples for Val shown in Fig. 4.

The assessment yielded a blank mass of 1.35 ± 0.41 µg C, with an associated F^14^C value of 0.33 ± 0.10. The low-blank mass suggests minimal contamination during sample preparation, and the F^14^C value indicates that the contaminant carbon is predominantly depleted in ^14^C. This finding demonstrates that ^14^C measurements on samples are feasible, as the level of contamination is sufficiently low to allow accurate ^14^C analysis.

### Problems/issues

During the testing of the apparatus, several issues were identified. Initially, we aimed to shift the cold fingertip by placing a 3D-printed plate between the cold finger base and the cap. This design was intended to disconnect the cold finger from the vacuum, preventing contamination of the capillary, while also acting as an insulating barrier between the hotplate and the cold finger. However, it became clear that maintaining a continuous connection to the vacuum pump was crucial for obtaining reliable and reproducible results. Additionally, heat transfer to the cold finger was found to be negligible at most operating temperatures, alleviating concerns about potential overheating.

Another issue involved the tolerance specifications of the GC vial threads, which were larger than those of the aluminum vial caps in our apparatus. This mismatch resulted in approximately 50% of the GC vials failing to establish a proper vacuum seal, with some vials being entirely misaligned with the caps. As a result, we had to manually check each vial for compatibility prior to use, adding an extra step to the process. The variations in GC vial threads also made it challenging to maintain a stable vacuum during sublimation runs, emphasizing the importance of continuous evacuation to minimize potential vacuum losses.

In one batch, ^14^C measurements were excluded from contamination assessment because the F^14^C values in several ^14^C modern Val samples were inexplicably elevated. While most affected samples were derived from prepared Val solutions, a few pre-weighed solid Val samples also exhibited elevated F^14^C values. Interestingly, none of the ^14^C dead samples showed any elevated ^14^C content. Furthermore, some solution-derived samples, particularly from later runs, displayed no elevated F^14^C values. Despite the anomalies, we used the results to evaluate reproducibility and recovery rates.

## Discussion

Microsublimation has proven to be a highly effective method for isolating vanillin from contaminants with significantly higher melting points, such as Val and Glu. The ^14^C measurements confirmed that these impurities did not co-sublime, even when present in significant quantities of over 20%. These findings demonstrate the robustness of microsublimation in maintaining sample purity during preparation, particularly when handling compounds with substantial differences in melting points.

In addition to high-boiling-point impurities, previous experiments (Heusser 2020) also showed that low-boiling-point contaminants can be effectively removed. In our current setup, such volatiles are typically eliminated either by vacuum application alone or in combination with mild heating prior to sublimation. Notably, the Peltier-cooled cold fingers can be gently preheated by reversing the current direction during the evacuation step, ensuring that no premature condensation or unintentional sublimation of volatile impurities occurs on the cold surfaces.

Our experiments on temperature dependence further show the suitability of microsublimation for amino acid and processing. By utilizing lower temperatures, particularly 200 °C for Val, we were able to avoid the risks associated with high-temperature sublimation, such as decomposition or impurity sublimation, as reported in previous work by Glavin and Bada (1998), Glavin et al. (2002), where temperatures up to 850 °C were used. Sublimating at 200 °C effectively preserved the physical and chemical properties of the amino acids while ensuring complete sublimation. These results highlight the efficiency of lower-temperature sublimation, especially when applied to a broad range of amino acids, without the need for extensive individual temperature optimization. Additionally, our design uses a simple experimental setup by relying on standard laboratory equipment. The controlled environment also ensures that impurities, like those seen in elevated-temperature processes, do not co-sublime, thereby maintaining sample purity.

The blank mass of 1.35 ± 0.41 µg C was notably low, demonstrating strong control over contamination during sublimation and confirming that contamination management was generally successful. In comparison, the glass prototype (Heusser 2020) exhibited higher constant contamination, with 2 µg C and an F^14^C of 0.45. This suggests that the lower contamination in the current analysis is likely due to reductions in surface area and the use of less material for the Al caps. However, the F^14^C value of 0.33 ± 0.10 still indicates the presence of ^14^C-depleted contaminant carbon, likely from residual solvents or trace impurities. The F^14^C values of modern Val samples from one batch (ETH nr. 134631.1.1–134677.1.1) had to be excluded due to inconsistent behavior, manifesting as elevated F^14^C values. While this reduced the number of data points, it ultimately improved the reliability of the dataset by removing anomalies.

The exclusion was necessary due to variability observed in modern Val samples, where some exhibited elevated ^14^C content while others did not. This suggests that the issue might be run-specific or linked to external environmental factors during sublimation. In contrast, dead samples consistently showed no elevated ^14^C content, reinforcing that the issue is confined to modern samples. The simultaneous occurrence of elevated F^14^C values in the syringic acid samples of the same batch further points to an external factor influencing these results. Additionally, one sample from another batch (ETH nr. 125312.1.1) also exhibited a high F^14^C value, also likely due to an external factor. These anomalous observations do not fit into existing contamination correction schemes (Haghipour et al. 2019; Sun et al. 2020; Hanke et al. 2017) and therefore meaningful treatment or correction of the data is currently unavailable. Although the specific factor responsible remains unidentified, controlling for this variable will be crucial to ensuring the accuracy of future experiments.

One possible source of contamination could be the formation of a thin aluminum oxide layer on the aluminum capsules. Exposure to air, particularly under humid conditions, could accelerate the formation of this oxide layer, which is known to adsorb atmospheric CO_2_ (Li et al. 2009). The drying process at 70 °C may have further facilitated this oxide formation. This layer could introduce variability in contamination assessments by adsorbing atmospheric gases, including CO_2_, contributing to the elevated F^14^C values observed in some samples. Further investigation into the extent of oxide layer formation and its impact on F^14^C levels is warranted to better understand and mitigate this potential source of contamination.

While contamination and equipment-related challenges posed obstacles, material-specific limitations also became apparent. Sublimation of *n*-alkanes revealed significant issues. Despite their relatively low melting points, both *n*-C_28_ and *n*-C_32_ exhibited low volatility, with minimal recovery at moderate temperatures. Significant sublimation only occurred at much higher temperatures (140 °C for *n*-C_28_ and 145 °C for *n*-C_32_), increasing the risk of co-sublimation of impurities. This demonstrates that sublimation is less suited to volatile compounds like *n*-alkanes, where maintaining purity becomes difficult. These limitations necessitate alternative methods when balancing purity with volatility.

In contrast, the sublimation of dye compounds yielded more promising results. Pure dyes, such as alizarin and purpurin, sublimed effectively at their respective melting points, highlighting the suitability of sublimation for isolating well-defined, single-component compounds. However, attempts to sublimate natural materials like madder root powder, which contains a complex matrix of anthraquinone glycosides, were unsuccessful. The presence of multiple compounds within the matrix likely interfered with the sublimation process, leading to charring and incomplete sublimation. This demonstrates that, while sublimation is effective for near-pure compounds, it struggles with complex natural matrices, where interactions between components can lead to decomposition. In such cases, compound-specific extraction and chromatographic purification would be required before applying microsublimation. Alternative purification methods may be required in these cases.

Further challenges were encountered with apparatus limitations. Issues such as the inability to maintain a stable vacuum due to the cold fingertip’s displacement from the vacuum seal highlighted design flaws. These concerns, however, were mitigated as it became evident that displacing the fingertip was unnecessary, and heat transfer to the cold finger was minimal. Additionally, the mismatch between GC vial threads and aluminum caps severely impacted vacuum stability, with half of the vials failing to establish a proper vacuum seal. Modifying the caps for better compatibility could resolve this, improving the reproducibility of the sublimation process.

The selection of compatible materials for sublimation-based techniques is therefore crucial. While microsublimation proved highly effective for isolating compounds with high-melting points, the results with *n*-alkanes and madder root demonstrate the limitations of this method for volatile substances and natural materials. In cases where sublimation is hindered by low volatility or complex matrices, alternative approaches must be considered to ensure both purity and recovery.

## Conclusions

The development of the aluminum microsublimation apparatus marks a substantial step forward in preparing ultra-small samples for CSRA. The combination of Peltier-cooled cold fingers, magnetically attached fingertips, and an efficient heat dissipation system provides a compact and modular setup that is robust, easy to operate, and delivers consistently low contamination levels. Blank measurements confirm that the apparatus enables accurate ^14^C analysis on small samples while overcoming many limitations of earlier glass-based prototypes.

Application tests demonstrate that microsublimation is particularly well suited for high-melting compounds such as amino acids, lignin phenols, and pure dyes, where high recoveries and clean separations were achieved. Conversely, volatile compounds like *n*-alkanes and complex natural matrices such as madder root proved less compatible, as recoveries were poor or degradation occurred. These results highlight both the strong potential of microsublimation for a broad set of target molecules and the need for alternative purification strategies when volatility or matrix effects dominate.

Remaining technical challenges mainly concern reproducibility and robustness of sealing, as well as occasional variability in contamination that may stem from material surface effects. Addressing these issues will further strengthen the method, but the present apparatus already establishes a reliable platform for solvent-free, low-blank purification. Its compact and modular design provides an adaptable foundation that could be readily implemented in other laboratories, supporting wider use of microsublimation in geochemical, archaeological, and environmental studies.

## Data Availability

The datasets used and analyzed during the current study are available from the corresponding author on reasonable request.

## Abbreviations

PCGC: Preparative capillary gas chromatography
HPLC: High-performance liquid chromatography
AMS: Accelerator mass spectrometry
CSRA: Compound-specific radiocarbon analysis
GC: Gas chromatography
EA: Elemental analyzer
MICADAS: Mini carbon dating system
